# The epidemiology of traumatic cervical spine fractures: a prospective population study from Norway

**DOI:** 10.1186/1757-7241-20-85

**Published:** 2012-12-21

**Authors:** Hege Linnerud Fredø, Syed Ali Mujtaba Rizvi, Bjarne Lied, Pål Rønning, Eirik Helseth

**Affiliations:** 1 Department of Neurosurgery, Oslo University Hospital–Ullevål, Oslo, N-0407, Norway; 2 Faculty of Medicine, University of Oslo, Oslo, Norway; 3 Department of Neurosurgery, Oslo University Hospital–Rikshospitalet, Oslo, Norway

**Keywords:** Cervical vertebrae, Spinal fractures, Trauma, Incidence, Epidemiology

## Abstract

**Aim:**

The aim of this study was to estimate the incidence of traumatic cervical spine fractures (CS-fx) in a general population.

**Background:**

The incidence of CS-fx in the general population is largely unknown.

**Methods:**

All CS-fx (C0/C1 to C7/Th1) patients diagnosed with cervical-CT in Southeast Norway (2.7 million inhabitants) during the time period from April 27, 2010-April 26, 2011 were prospectively registered in this observational cohort study.

**Results:**

Over a one-year period, 319 patients with CS-fx at one or more levels were registered, constituting an estimated incidence of 11.8/100,000/year. The median age of the patients was 56 years (range 4–101 years), and 68% were males. The relative incidence of CS-fx increased significantly with age. The trauma mechanisms were falls in 60%, motorized vehicle accidents in 21%, bicycling in 8%, diving in 4% and others in 7% of patients. Neurological status was normal in 79%, 5% had a radiculopathy, 8% had an incomplete spinal cord injury (SCI), 2% had a complete SCI, and neurological function could not be determined in 6%. The mortality rates after 1 and 3 months were 7 and 9%, respectively. Among 319 patients, 26.6% were treated with open surgery, 68.7% were treated with external immobilization with a stiff collar and 4.7% were considered stable and not in need of any specific treatment. The estimated incidence of surgically treated CS-fx in our population was 3.1/100,000/year.

**Conclusions:**

This study estimates the incidence of traumatic CS-fx in a general Norwegian population to be 11.8/100,000/year. A male predominance was observed and the incidence increased with increasing age. Falls were the most common trauma mechanism, and SCI was observed in 10%. The 1- and 3-month mortality rates were 7 and 9%, respectively. The incidence of open surgery for the fixation of CS-fx in this population was 3.1/100,000/year.

**Level of evidence:**

This is a prospective observational cohort study and level II-2 according to US Preventive Services Task Force.

## Background

The incidence of traumatic cervical spine fractures (CS-fx) in the general population is largely unknown. Several reports describe the incidence of CS-fx in different subpopulations, such as trauma center patients, specific age groups, head injury patients, military populations and osteoporotic patients [1-15]. Our literature search identified only one article describing the incidence of spine fractures in a general population. In 1996, Hu et al. reported the incidence of spine fractures, all sites, to be 64/100,000 [16]. However, subgrouping into cervical, thoracic or lumbar fractures was performed for only 45% of the patients. The assumed incidence of CS-fx can be estimated to be 12/100,000 based on this study, which was performed on a population in Canada.

CS-fx are more common in males than females [1,4-6,10,11,13,17-21]. With respect to age, the highest incidence rate is reported to be among patients aged 15–45 years, with a second peak in those aged 65 – 80 years [1,4-6,17,21-24]. Most articles report motor vehicle collisions to be the most frequent trauma mechanism leading to cervical spine injury, [1,2,13,14,19,21] while a minority report falls as the most frequent [10,22,23]. The incidence of cervical spine injury in the setting of head injury has been reported to range between 1.8-9% [4,7-9,14,15,18,25]. Conversely, the incidence of moderate to severe head injury in patients with cervical spine injury is reported to be between 18 – 40% [1,7-9,22,23,26]. Spinal cord injury (SCI) is reported to occur in 12 – 50% of patients with CS-fx [4,9,11,14,16,17,21-23]. The relationships described between CS-fx and gender, age, trauma mechanism, head injury and SCI are based on studies of subpopulations of trauma patients and not on trauma cases from a general population. Thus, these “well-accepted” relationships may not be true for cervical spine injuries in a general population.

The main aim of this study was to determine the incidence of CS-fx in a general population. We also sought to investigate possible risk factors for CS-fx, such as gender, age and trauma mechanism. In addition, the frequencies of neurological deficit and open surgical fixation in these patients were studied.

## Methods

Oslo University Hospital-Ullevål (OUS-U) is a level 1 trauma center situated in Oslo. It is the only neurosurgical trauma center for the Southeast part of Norway (120,000 km^2^), which has 2.70 million inhabitants. The age distribution in this population is as follows: 541,077 aged 0 – 15 years, 524,546 aged 16 – 30 years, 578,001 aged 31 – 45 years, 520,114 aged 46 – 60 years, 358,150 aged 61 – 75 years, 162,750 aged 76 – 90 years and 15,353 aged >90 years. OUS-U performs >95% of the trauma-related neurosurgical procedures in this population, including all surgeries for cervical spine injury. There are 20 hospitals within our region with general and/or orthopedic surgeons and radiological services that refer patients with head and cervical spine injuries to us. The patients are either admitted to OUS-U for treatment or non-surgical treatment is carried out locally after consultation with the Department of Neurosurgery at OUS-U.

To determine the incidence of CS-fx in this prospective observational cohort study, we prospectively registered all CS-fx patients (C0/C1 to C7/Th1) diagnosed with cervical-CT (frequently supplemented with cervical MRI) in Southeast Norway from April 27, 2010-April 26, 2011.

The following data were recorded: sex, age, anatomical level of injury, number of cervical levels injured, trauma mechanism according to ICD-10, [27] fall injuries subclassified according to the Canadian C-spine rule [12] based on the mechanism of injury (falls from ≥1 meter or ≥5 stairs) (yes/no), Head Injury Severity Score (HISS), [28] ankylosing spondylitis (yes/no), concomitant thoracolumbar fracture (only patients admitted to OUS-U) (yes/no), neurological deficit at the time of diagnosis (normal, radiculopathy, incomplete spinal cord injury (SCI), complete SCI or unknown (due to severe head injury or extensive multitrauma)), primary treatment (none, external immobilization with stiff collar, open surgical fixation) and mortality 1 and 3 months after the diagnosis. Vital status (dead or alive) and time of death were determined based on the Norwegian Population Registry (Folkeregisteret) data for August 1. 2011. The two major groups of fractures were further classified, odontoid fractures according to Anderson and D’Alonzo [29] and subaxial fractures according to the Subaxial Injury Classification system (SLIC) [30].

The SLIC system is based on literature review and expert opinions, designed to describe and classify cervical spine injury. It takes into account 3 major aspects of injury; the morphology, the disco-ligamentous complex and the neurological status. In each category, points are awarded for abnormalities, more points indicate a more severe injury, and the total score leads to a recommendation for surgical or non-surgical treatment.

We registered all patients diagnosed in our health region, including patients who were just visiting on vacation or work immigrants without a Norwegian social security number. The number of patients without a Norwegian social security number was 17 (5.3%): 11 were working in Norway, three were on vacation and three could not be located.

## Results

In our defined population of 2.7 million people (Southeast Norway), we prospectively registered 319 patients with one or more CS-fx during the year of registration. This registration yielded an incidence of CS-fx of 11.8/100,000.

**Figure 1:**
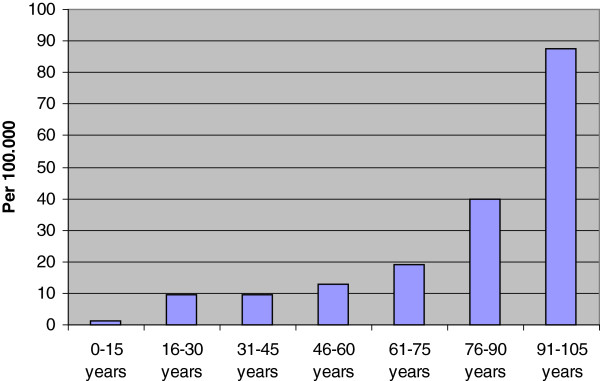
Age-stratified incidence of CS-fx.

The median age of the patients was 56 years (range 4–101 years), and 217/319 (68%) were males (Table 1). The mean age of males was significant lower than for females (p = 0.01). The relative incidence of CS-fx increased significantly with age (Table 2 and Figure 1).

**Table 1:** Patient characteristics

		**N (%)**
**All**		319 (100)
**Sex**	Female	102 (32.0)
	Male	217 (68.0)
**Age group**	0-15	7 (2.2)
	16-30	49 (15.4)
	31-45	56 (17.6)
	46-60	65 (20.4)
	61-75	64 (20.1)
	76-90	65 (20.4)
	91-105	13 (4.1)
**Ankylosing spondylitis**	Yes	15 (4.7)
**Number of levels injured**	1	237 (74.3)
	2	68 (21.3)
	3	10 (3.1)
	4	3 (0.9)
	5	1 (0.3)
**C0–C7 fx**	Total	420 (100)
**C0–C2 fx**	C0	22 (5.2)
	C1	27 (6.4)
	C2 + C2/3	98 (23.3)
**C3–C7 fx**	C3 + C3/4	25 (6.0)
	C4 + C4/5	39 (9.3)
	C5 + C5/6	61 (14.5)
	C6 + C6/7	89 (21.2)
	C7 + C7/Th1	59 (14.0)
**Neurological status at the time of diagnosis**	Normal	252 (79.0)
	Root injury	15 (4.7)
	Incomplete SCI	27 (8.5)
	Complete SCI	6 (1.9)
	Unknown	19 (6.0)
**HISS**	No head injury	34 (10.7)
	Minimal	181 (56.7)
	Mild	69 (21.6)
	Moderate	13 (4.1)
	Severe	22 (6.9)

**Table 2:** Age-stratified incidence rate of CS-fx in the Southeastern part of Norway with 2.70 million inhabitants

**Age group**	**Cs-fx (N)**	**Population**	**Incidence**	**95% CI**
0–15 years	7	541,077	1.9	0.52–26.66
16–30 years	49	525,546	9.3	6.89–123.35
31–45 years	56	578,001	9.7	7.31–125.85
46–60 years	65	520,114	12.5	9.64–159.34
61–75 years	64	358,150	17.9	13.76–228.19
76–90 years	65	162,750	39.9	30.79–509.22
91–105 years	13	15,353	84.7	45.05–1,448.77

The trauma mechanisms were falls in 60%, motorized vehicle accidents in 21%, bicycling in 8%, diving in 4% and others in 7% (Table 3). Fall-related injuries were subclassified according to the Canadian C-spine rule, which identified falls from ≥1 meter or ≥5 stairs to be responsible for the injuries among 82 patients (43%). Patients with fall-related injuries tended to be older, while patients injured in motorized vehicle-, bicycle- or diving accidents tended to be younger (Tables 3 and 4).

**Table 3:** Mechanism of trauma

**ICD-10**	**N (%)**	**Mean age (range)**	**Males N (%)**
Falls	190 (59.6)	65 (12–101)	124 (65)
*W0n7p*, *W0n0r*, *W0n9r*, *W0n5r*, *W0n2r*, *W0n9a*, *W0n9b*, *W0n6r*, *W0n2h*			
Motorized vehicles	67 (21.0)	41 (5–88)	44 (66)
*V2n1r*, *V4n1r*, *V6n2r*, *V2n7p*, *V2n2r*, *V5n2k*			
Bicycle	25 (7.8)	48 (22–73)	17 (68)
*V1n2p*, *V1n1r*			
Pedestrian	4 (1.3)	45 (37–90)	3 (75)
*V0n1r*, *V888r*			
Diving	13 (4.1)	33 (14–55)	13 (100)
*W0n8r*			
Other	20 (6.3)	41 (4–94)	16 (80)
*X59xy*, *X8n9r*, *W7n0r*, *W2s9b*, *W2s1r*, *W2s0r*, *W2s8p*, *W237p*, *X6n0y*, *X8n0r*, *W2s7p*, *W2s1p*, *W23xy*, *W231r*			
	319 (100%)	55 (4 – 101)	217 (68)

**Table 4:** Mechanism of trauma versus age groups

		**ICD-10 group**						**Total**
		**Falls**	**Motorized vehicles**	**Bicycle**	**Pedestrian**	**Diving**	**Other**	
AgeGroups	0-15 years	3	1	0	0	1	2	7
	16-30 years	16	21	3	0	4	5	49
	31-45 years	19	15	9	2	6	5	56
	46-60 years	40	9	9	1	2	4	65
	61-75 years	48	10	4	0	0	2	64
	76-90 years	52	11	0	1	0	1	65
	91-105 years	12	0	0	0	0	1	13
Total		190	67	25	4	13	20	319

Among 319 patients, a total of 420 injured levels were identified; 81 patients (26%) had fractures at more than one cervical level (Table 1). There were 147 high CS-fx (C0-C2) and 273 subaxial (C3-C7) fractures. The levels affected most frequently were C2 and C5-7. A combined high- and subaxial fracture was observed in 17 patients. The median age for patients with C0-C2 fractures was 66 years (range 16–101), and 58% were males. The median age of patients with subaxial fracture was 49 (range 4–94), and 74% were males.

Ultimately, 228 of the 319 patients included in this study were admitted to OUS-U. In this subgroup, 50 (22%) patients had a concomitant fracture in the thoracolumbar spine.

SCI was present in 33 patients (10%) (Table 1). Neurological disability was significantly more frequent in subaxial fractures than in C0-C2 fractures (Table 5). SCI tended to be more frequent in younger patients (Table 6).

**Table 5:** Neurological status at time of diagnosis for patients with C0–C2 and subaxial fractures (fxs)

**Neurological Status**	**C0–C2 fx (N = 133) N (%)**	**Subaxial fx (N = 203) N (%)**	**Fisher’s exact test**
Normal	117 (88)	152 (75)	*p* < 00.1
Root	0 (0)	15 (7)	
Incomplete SCI	5 (4)	22 (11)	
Complete SCI	0 (0)	6 (3)	
Unknown	11 (8)	8 (4)	

17 of the 319 patients included in this study had both C0-C2 and Subaxial Fractures (fxs). Thus, the sum of C0-C2 and Subaxial fxs in this table adds up to 336. Regarding the 17 patients with combined fxs, the charts/MRI scans were used to determine wheter the neurological deficit(s) were associated with the axial or subaxial components of the injury.

**Table 6:** Neurological status versus age groups

		**Neurological status**					**Total**
		**Normal**	**Root**	**Incomplete SCI**	**Complete SCI**	**Unknown**	
Age Groups	0-15 years	6	0	0	0	1	7
	16-30 years	39	3	1	2	4	49
	31-45 years	41	5	5	3	2	56
	46-60 years	51	5	4	0	5	65
	61-75 years	47	2	10	1	4	64
	76-90 years	57	0	5	0	3	65
	91-105 years	11	0	2	0	0	13
Total		252	15	27	6	19	319

### Treatment

Ultimately, 85 (26.6%) of the patients were treated with open surgery, while 219 (68.7%) were treated with external immobilization with a stiff collar, and 15 (4.7%) were considered stable and not in need of any specific treatment. These results yield an estimated incidence for surgically treated CS-fx in our population of 3.1/100,000/year. The data showed that 17/133 (12.8%) patients with C0 – C2 fractures and 68/203 (33.5%) with subaxial fractures underwent surgery.

A total of 61 odontoid fractures were identified. According to the Anderson and D’Alonzo classification [29] there were 35 type 2 and 26 type 3 fractures. Eleven of the odontoid fractures (18%) were treated with open surgery; of these, 7 patients had type 2 fractures and 4 patients had type 3 fractures.

All 203 patients with subaxial injuries were classified according to SLIC [30]. Open surgical fixation was performed in 68/203 (33.5%) patients. Of 68 patients with SLIC scores ≥5, 55 (81%) were treated with open surgery and 13 (19%) with external immobilization. Of 27 patients with SLIC scores = 4, 8 were treated with open surgery and 19 were treated with a stiff collar. Of 108 patients with SLIC scores <4, 5 were treated with open surgery, 94 were treated with stiff collars, and nine patients received no treatment.

### Mortality

The 1- and 3-month mortality rates in this series were 7 and 9%, respectively. For surgically treated patients, the 1- and 3-month mortality rates were 2% and 3%, respectively. The 3-month mortality was 15% for patients with C0-C2 fractures and 6% for patients with subaxial and combined axial and subaxial fractures.

## Discussion

This prospective study estimates the incidence of traumatic CS-fx in a general Norwegian population to be 11.8/100,000/year. The incidence of open surgery for the fixation of CS-fx in this population is 3.1/100,000/year. Thus, 27% of the CS-fx were treated by open surgery.

We identified only one previous publication from which we could estimate the incidence of traumatic CS-fx in a general population. This Canadian study from 1996 reported the incidence of spine fractures at all sites to be 64/100,000/year [16]. Subgrouping into cervical, thoracic or lumbar fractures was performed for only 45% of the patients that were admitted to hospitals. In these patients, the site involved was cervical in 19% of patients, thoracic in 30% of patients, lumbosacral in 43% of patients, and unspecified in 8% of patients. If we assume fractures at all levels were admitted to the same extent, this would give an incidence of CS-fx in this population of 12/100,000/year. This incidence number is almost identical to what we find in our population.

Other authors report higher incidence numbers, but these populations are subgroups, similar to the large military population described by Schoenfeld, who found the incidence of traumatic CS-fx to be 29/100,000/year [17].

We observed a male predominance among our patient population. This is in accordance with previous reports [1,4-6,10,11,13,17-21]. The age distribution in our series reveals the highest frequency of cervical spine fracture among those aged 46–90 years. When adjusted for the age distribution in the Norwegian population, we found a steadily increasing incidence with rising age. This is in contrast to former reports, which found the third decade of life or age between 15–45 years to be more strongly associated with an increased risk of cervical spine injury [1,4,5,17,21,22,24]. Some authors also reported a second “peak” at age 65–80, [6,23,24] which is more in accordance with our results. We found a low incidence of cervical spine fractures in children, as reported by others [2,3,6,15,23].

Fall-related injuries were observed in more than half of our patients. Most other authors have found motor vehicle collisions to be the most frequent trauma mechanism leading to cervical spine injury, with falls the second most frequent [1,2,13,14,19,21]. A minority of authors report the same findings that we do regarding trauma mechanism [10,22,23]. Minimally dangerous situations (falls from <1 meter or <5 stairs) accounted for 57% of the falls in our material. It is probable that these injuries are frequently not included in subgroups of trauma center populations. These patients are often referred from general practitioners or emergency primary care because of sustained neck pain after a minor trauma. Some recent reports indicate a trend during recent decades of an increasing median age of spine trauma patients and a shift towards falls as the most frequent mechanism of injury [19,20]. Our results are in line with this trend.

The incidence of cervical spine injury in patients with moderate and severe head injury has been reported to range between 1.8% and 9% [4,7-9,14,15,18,25,31]. Some authors, though not all, report that patients in a trauma population with clinically significant head injuries are at greater risk of cervical spine injury than those without head trauma and that patients with a severe head injury (GCS ≤8) are at even greater risk. Conversely, the incidence of moderate to severe head injury in patients with cervical spine injury is reported to be 18 – 40% [1,7-9,22,23,26]. In our series, 11% of the patients had a concomitant moderate or severe head injury. The lower rate of moderate and severe head injuries observed in our material is most likely because we have studied a general population and not a selected subpopulation. In contrast to many reports from selected trauma populations, we also include the low-energy trauma that often leads to cervical spine fracture in the elderly. However, the rates of minimal and mild head injury in our patients were as high as 78%, so there is no doubt that head and neck injuries are closely associated.

In our patients with cervical fractures, neurological status was normal in 79%, 5% had a radiculopathy, 8% had incomplete SCI, 2% had complete SCI and neurological function could not be determined by chart review in 6%. Neurological disability was significantly more frequent in subaxial fractures than in C0-C2 fractures. The incidence of cervical SCI in the setting of a cervical spine fracture is reported to be 12-50% [4,9,11,14,16,17,21-23]. Only 10% of the patients in our study population had SCI. Again, the lower rate of SCI in our report compared to other series is likely due to our study of the general population rather than a subpopulation. The incidence of cervical SCI in the setting of a CS-fx is 1.2/100,000/year.

In a population study from Western Norway, the incidence of acute traumatic SCI during the time period from 1997–2001 was estimated to be 1.4/100,000 [32]. This study also included SCI without concurrent CS-fx.

The 1- and 3-month mortality rates in this series were 7 and 9%, respectively. For surgically treated patients, the 1- and 3-month mortality rates were 2% and 3%, respectively.

We have not found comparable mortality data on patients with CS-fx from a general population, but according to the mortality rates reported in different subgroups, the mortality in our population seems to be in the lower range [1,4,22,33-35].

Among all 319 patients, 27% were treated with open surgery, 68% were treated with external immobilization with a stiff collar and 5% were considered stable and not in need of any specific treatment. This distribution yields an estimated incidence of surgically treated CS-fx in our population of 3.1/100,000/year.

Of the odontoid fractures, 18% were treated with open surgery, seven were type 2 fractures and four were type 3 fractures. There is still insufficient evidence to establish a standard or guideline for odontoid fracture management, and there are various reported approaches for treatment [36-38].

Open surgical fixation was conducted in 33.5% of the patients with subaxial fractures. Among patients with SLIC scores ≥5, 81% were treated with open surgery and 19% with external immobilization. These are cases in which the SLIC recommended open surgical treatment. Among the population studied here, 37% of the patients with SLIC scores = 4 underwent open surgery, and 63% received a stiff collar. In this group, SLIC found the treatment options to be equivocal. The records showed that 5% of the patients with SLIC scores <4 were treated with open surgery, 87% were treated with stiff collars, and 8% received no treatment. The SLIC recommendation for these patients is non-surgical treatment. In this investigation, 9% of our patients with subaxial CS-fx were not treated with the modality recommended based on the SLIC score. However, concomitant and comorbidity factors are to be taken into consideration when selecting treatment, and these factors are not accounted for in the treatment algorithms used here. We therefore find our compliance with the SLIC recommendations to be acceptable.

### Limitations

The incidence of CS-fx found in our study is most likely an underestimate because we believe that some fractures are considered stable at their local orthopedic departments, and they are treated with external immobilization without our knowledge. We know this for certain because some of these patients are later referred to us when the non-surgical treatment has failed. Data from The Norwegian Patient Registry also support the assumption that the present data are an underestimate (HL Fredø et al., unpublished results). Some fractures causing immediate death at the scene of the accident are also missed when using this approach. Reports suggest that 21-24% of victims dying immediately or soon after a traffic accident have a serious injury to the cervical spine [39,40].

### Strengths

Our data were registered prospectively in a defined general population, and the study is contemporary. All charts and radiological examinations were reviewed.

## Conclusions

This prospective study estimates the incidence of traumatic CS-fx in a general Norwegian population to be 11.8/100,000/year. A male predominance was observed, and the incidence increased with advancing age. Falls were the most common trauma mechanism, and SCI was observed in 10% of those included. The 1- and 3-month mortality rates were 7% and 9%, respectively. The incidence of open surgical fixation of CS-fx in this population is 3.1/100,000/year.

## Competing interests

The authors declare that they have no conflict of interest. The authors alone are responsible for the content and writing of this paper.

## Authors’ contribution

Study design: HLF and EH. Collection of data: HLF and SAMR. Data analysis and statistics: HLF, SAMR, BL, PR and EH. Interpretation of data: HLF, SAMR, BL, PR and EH. Writing of manuscript: HLF, PR and EH. All authors read and approved the final manuscript.
